# Realistic 3D Phantoms for Validation of Microwave Sensing in Health Monitoring Applications

**DOI:** 10.3390/s24061975

**Published:** 2024-03-20

**Authors:** Mariella Särestöniemi, Daljeet Singh, Rakshita Dessai, Charline Heredia, Sami Myllymäki, Teemu Myllylä

**Affiliations:** 1 Health Sciences and Technology, Faculty of Medicine, University of Oulu, 90220 Oulu, Finland; daljeet.singh@oulu.fi (D.S.); teemu.myllyla@oulu.fi (T.M.); 2 Centre for Wireless Communications, Faculty of Information Technology and Electrical Engineering, University of Oulu, 90570 Oulu, Finland; 3 Microelectronics Research Unit, Faculty of Information Technology and Electrical Engineering, University of Oulu, 90570 Oulu, Finland; rakshita.dessai@oulu.fi (R.D.); sami.myllymaki@oulu.fi (S.M.); 4 Optoelectronics and Measurements Research Unit, Faculty of Information Technology and Electrical Engineering, University of Oulu, 90570 Oulu, Finland; heredia.charline@gmail.com; 5 Medical Research Center Oulu, 90014 Oulu, Finland

**Keywords:** digital twins for healthcare, medical monitoring, human tissue phantoms, microwave technology, phantom verification

## Abstract

The development of new medical-monitoring applications requires precise modeling of effects on the human body as well as the simulation and the emulation of realistic scenarios and conditions. The first aim of this paper is to develop realistic and adjustable 3D human-body emulation platforms that could be used for evaluating emerging microwave-based medical monitoring/sensing applications such as the detection of brain tumors, strokes, and breast cancers, as well as for capsule endoscopy studies. New phantom recipes are developed for microwave ranges for phantom molds with realistic shapes. The second aim is to validate the feasibility and reliability of using the phantoms for practical scenarios with electromagnetic simulations using tissue-layer models and biomedical antennas. The third aim is to investigate the impact of the water temperature in the phantom-cooking phase on the dielectric properties of the stabilized phantom. The evaluations show that the dielectric properties of the developed phantoms correspond closely to those of real human tissue. The error in dielectric properties varies between 0.5–8%. In the practical-scenario simulations, the differences obtained with phantoms-based simulations in *S21* parameters are 0.1–13 dB. However, the differences are smaller in the frequency ranges used for medical applications.

## 1. Introduction

New wearable and portable medical monitoring and screening techniques are being investigated for future wireless healthcare and telemedicine applications. The development and optimization of these emerging techniques require numerous trials and rounds of experimentation before the techniques can reach the standards required for clinical applications. These trials are generally performed on humans or animals such as rats, pigs, or monkeys. This process proves to be very costly in terms of time and money and is often not very straightforward due to ethical issues. Therefore, the development of realistic simulation and emulation platforms is essential for evaluating these techniques in their initial phase of development. In recent years, the research community has shown a keen interest in the development of more accurate and realistic phantoms for different sensing techniques based on microwaves, magnetic resonance imaging (MRI), optics, and acousto-optics. Emulation platforms are usually built with human-tissue-mimicking phantoms [1,2,3,4,5,6,7,8,9,10] or with animal tissues, e.g., pig tissues [11,12,13,14]. This paper focuses on phantom development for microwave techniques.

### 1.1. Microwave Sensing and Its Advantages

Microwave sensing is recognized as one of the most promising emerging techniques for medical monitoring and screening applications due to its several advantageous features. Some of these features include good resolution, low power, low cost, reliability, security, and sufficient propagation depth, which enables deep-tissue monitoring. Especially at lower frequency bands (1–4 GHz), superior propagation depth can be achieved using microwaves compared with other modalities. Microwaves are considered suitable for deep-tissue-monitoring applications that require greater penetration depth, such as whole-brain screening and breast-health monitoring, as well as for implant communications, such as capsule endoscopy.

Studies in the literature propose that a better resolution can be achieved with microwaves at higher frequencies as a result of a specific correlation between water content and electromagnetic field at such high frequencies. During the past decade, several microwave techniques-based studies have been proposed for different medical and healthcare applications [15,16,17,18,19,20]. Some of these studies have also focused on the development of phantom models for microwave systems. An overview of the development of state-of-the-art tissue-mimicking phantoms for different microwave technique-based applications is presented in the following section.

### 1.2. State-of-the-Art Tissue-Mimicking Phantom Development for Micalrowaves

The characteristics of phantoms enable the quantification of the state, shape, and operational mechanisms of the actual human body without the fuss and risk of using actual human subjects. Furthermore, phantoms have many advantageous features such as a fast construction process, low cost, repeatability, and reusability, which make them an even more suitable option than human and animal trials. Due to all these reasons, many different tissue-mimicking (TM) phantom models have been proposed in the literature to simulate different organs/parts of the human body. These phantoms can be categorized into several classes based on the materials used (solid, liquid, hybrid), number of layers, purpose (sensing, imaging, detection), and level of detail in their architecture. The design of a good phantom for microwave studies essentially requires the phantom to mimic not only the shape and physical properties of the original biological tissue, but also to precisely replicate its dielectric properties. However, a phantom model that includes very fine details of the tissue requires an intricate design that is challenging to manufacture and reproduce. Therefore, a majority of the phantom models proposed in the literature are very simplistic and are created based on crude assumptions. The following subsections present different phantom models for modeling the brain and breast.

#### 1.2.1. Brain Phantoms

In [21], a six-layered human-head phantom was proposed for use in a sensor-based microwave brain imaging system (SMBIS) for the diagnosis of tumors in the head. The phantom model consisted of dura, CSF, white matter, gray matter, fat, and skin, and was intended for a wideband frequency band (1 GHz to 4 GHz). A tissue-mimicking 3D head phantom was fabricated with dura, CSF, gray matter, white matter, and blood (hemorrhage) agar, gelatin, distilled water, corn flour, propylene glycol, sodium azide, and sodium chloride in [22]. The presented phantom was tested for a frequency range of 1 GHz to 4 GHz.

Karadima et al. [23] proposed a brain-phantom model for the validation of microwave tomography with the DBIM-TwIST algorithm for a frequency range of 0.5 to 2.5 GHz. The phantom consisted of an average brain, CSF/blood, and ischemia layers made from gelatine power, kerosene, safflower oil, propanol, and surfactant. The effect of aging on the dielectric properties of phantoms was also studied. In [24,25] a head phantom to test microwave systems for brain imaging was proposed and tested for 1–4 GHz. The brain phantom consisted of CSF, gray and white matter, and blood. The dielectric properties of the fresh sample and the sample after four weeks of aging were presented. The recipe for the proposed phantom included water, corn flour, gelatin, agar, sodium azide, and propylene glycol as the main components.

The heterogeneous brain-phantom model presented by Najafi et al. [26] used polytetrafluoroethylene and methyl methacrylate as bone and soft tissue, and the phantom was tested for stereotactic radiosurgery. Joachimowicz et al. [27] proposed the use of TX-100 and salted water for the preparation of a brain phantom for a frequency range of 0.5–6 GHz. The phantom consists of the brain, CSF, muscle, bone, and blood made by mixing TX-100 and NaCl in different proportions. Pokorny et al. [28] proposed an anatomically and dielectrically realistic 2.5 D give-layer reconfigurable head phantom. The phantom consists of five different layers that mimic the scalp, skull, cerebrospinal fluid, brain, and stroke regions and are synthesized from urethane rubber, graphite powder, carbon black powder, and acetone. The liquid brain consists of a solution of deionized water, isopropanol, and NaCl.

Mobashsher and Abbosh [29] proposed a human head phantom wherein the skull cavity was constructed using 3-D-printed molds representing gray matter, white matter, dura, CSF, eye, cerebellum, spinal cord, and blood. The phantom was tested at a frequency range of 0.5–4 GHz, and water, corn flour, gelatine, agar, sodium azide, propylene glycol, and NaCl were utilized in the construction of the phantom model. Another human brain phantom was proposed in [30] by Suleiman et al. for a frequency range of 1 to 4 GHz. The phantom was constructed using appropriate combinations of water, corn flour, gelatin, agar, sodium azide, and propylene glycol, and dielectric properties from [31] were taken as references for preparing the phantom. The measurements reported in [30] were repeated two months after phantom creation to confirm the stability of the properties over time.

Luis et al. [32] proposed a brain phantom consisting of brain, fat, muscle, gray matter, white matter, and blood. The bone was made of plaster, water, and ethyl alcohol was used for muscle, while the gray matter was composed of water, sugar, NaN3, and agar. The white matter of the phantom was constructed using water and ethyl alcohol, while the blood sample was created using a mixture of water, sugar, NaN3, and agar. Another phantom model was proposed in [33] by Konstantinos et al. and tested for four frequency bands of 1.1 GHz, 1.8 GHz, 2.4 GHz, and 2.8 GHz. Two cylindrical containers with radii of 7 cm and 2 cm were utilized for the phantom. Deionized water at room temperature and tepid deionized water were poured into the two containers to mimic the dielectric properties of the brain during the experiments.

A brain phantom for the ISM 2.4-GHz band was proposed in [34], wherein a 4-mm-thick skin was used around the phantom with 10-mm-thick cortical bone. Further, the phantom model consisted of gray and white matter and muscle. The conductivity and relative permittivity were measured and found to match the actual biological brain. A polyvinyl chloride (PVC) based head phantom was proposed in [35] by Mohammed et al. which consists of soft brain tissues with main ingredients as propylene glycol, water, grape seed oil, commercial dishwashing liquid, sodium azide, corn flour. Authors in [36] used the recipe from [29] to fabricate a five-layer human brain phantom consisting of dura, CSF, gray matter, white matter, and blood for hemorrhage. The basic materials such as Agar, gelatin, corn flour, sodium azide, sodium chloride, distilled water, and propylene glycol were utilized in making this phantom model.

A skull phantom filled with liquids and semi-solids was proposed in [37] for brain hemorrhage studies. The thickness of the skull bone was taken to be 7 mm, and the recipe consisted of a mixture of epoxy, SrTiO_3_ powder, ethanol, distilled water, glycerol, sucrose, and salt. Shahidul et al. [38] proposed a phantom model with CSF, dura, gray matter, white matter, and blood for 1–4 GHz. Sterile water, corn flour, gelatin, agar, sodium azide, propylene glycol, and sodium chloride were used in the construction of different parts of the phantom. Jacob et al. [39] proposed a four-layer phantom model with skin, skull, cerebrospinal fluid, and brain for 2–3 GHz frequency. The thickness of the skin was 5 mm, the CSF was kept at 10 mm, and the brain was 5 mm, with agar, polyethylene powder (PEP), TX-151, and sodium chloride as main ingredients. A brain-phantom model based on a 3D-printed structure was presented in [40], wherein the biological tissues of the brain were mimicked by a liquid solution of TX-100 and NaCl.

#### 1.2.2. Breast Phantoms

Joachimowicz et al. [41] proposed a breast phantom for microwave imaging in the 0.5–6 GHz range using the one-pole Debye model. The effect of time and temperature on the stability of the proposed phantom was studied. Porter et al. developed a realistic breast phantom for breast-cancer detection using n-propanol, deionized water, bloom gelatin, formaldehyde, oil, and Ivory Ultra detergent [8]. The phantom consists of skin, fat, small glands, and large glands and is intended for use in tumor detection. A similar oil-in-gelatin recipe was utilized in four heterogeneous breast phantoms in [42]. The phantoms were constructed in order to cover the complete range of volumetric breast densities for microwave-imaging experiments. Homogenous as well as heterogeneous realistic breast phantoms composed of skin, fat, glandular tissue, and tumors were presented by Islam et al. in [43]. The proposed phantoms were tested for a 3.1–10.6 GHz frequency range.

Even though these oil-in-gelatin phantoms can replicate the dielectric properties of actual breast tissues, they are hypersensitive to environmental exposure, and their properties can deteriorate with time. Therefore, liquid-based phantoms consisting of mixtures of distilled water and polyethylene glycol mono phenylether (Triton X-100) were proposed in literature. These phantoms are easy to generate and conserve over time [44]. Gunnarsson et al. also proposed a liquid-based breast phantom model using Triton X-100, water, and salt mixtures [45,46,47]. A study on Triton X-100 and distilled water-based phantom models for 0.5–12 GHz frequency band was presented in [48]. The phantoms were tested for their temperature stability in the range of 18–30 degree Celsius. The authors of [49] presented an interesting survey of numerical breast phantoms based on software and physical phantom models. Garrett and Fear presented a breast phantom made from carbon and rubber [50]. The phantom model was generated using a 3D-printing technique and consisted of skin, fat, glandular tissue, and tumor tissue.

### 1.3. Objectives and Novelty of This Study

This paper is an extension of and complement to our previous studies [15,51,52]. The first objective of this paper is to present a new approach for developing and evaluating realistic emulation platforms for human head and torso areas. The main focus is on 3D phantom emulation platforms for (a) brain-tumor/stroke detection, (b) breast-cancer detection, and (c) abdominal-disorder detection e.g., using capsule endoscopy. The paper describes phases of phantom development including several new recipe trials, the suitability of which is verified by measurement of the phantoms’ dielectric properties.

The second objective of this paper is to present a new method to verify the feasibility and reliability of the phantoms for use in practical scenarios using electromagnetic simulations to calculate antenna reflection coefficients and channel parameters, with the tissue-layer simulations having the same dielectric properties as the developed phantoms. The results are compared using a reference-layer model with the dielectric properties of realistic human tissues. The third objective is to study the impact of phantom cooking temperature on the dielectric properties of the final phantom.

This paper is organized as follows: Section 2 presents the Materials and Methods, which consists of materials and procedures for the development of 3D molds and phantoms. Additionally, simulation models used in phantom verification are shown. The measurement systems used to quantify dielectric properties and S-parameters are explained. The results for dielectric property measurements and phantom verifications with electromagnetic simulations are presented in Section 3. The discussion and plans for future work are presented in Section 4.

## 2. Materials and Methods

It is crucial to use multilayer models instead of averaged models during evaluations for practical applications. This section presents the material and methods utilized in this study.

### 2.1. Materials Used in Phantom Development

The characteristics of human tissues depend primarily on their water content, as visualized in Table 1 [53]. Human-tissue-mimicking phantoms were prepared using ingredients and materials that are easily accessible in supermarkets, pharmacies, or chemical stores. The primary materials utilized for phantom preparation were distilled water, gelatine, sunflower oil, sugar, sodium chloride (NaCl), xanthan, and propylene glycol (pure, 98%).

The recipes for different human tissues consist of varying amounts of these ingredients, as shown in Table 2. Recipes 1–5 are improved, modified versions of recipes presented in several sources from the literature. The muscle and intestinal recipes are derived from [6], and the fat phantom recipe is a novel proposal developed by us in [51], with ingredients not used in previous recipes. It is interesting to note that the dielectric properties of actual human tissue exactly match those of the phantoms formulated in this manuscript. The values were verified from the literature [6,51,53].

### 2.2. Procedure to Prepare Phantoms of Different Tissues

This section describes the details of the procedures used to prepare different tissue-phantom recipes. The description is divided into four different categories, with the category depending on the ingredients used (a) brain, skin, and tumor 1 phantoms; (b) glandular phantom; (c) muscle phantom; (d) fat phantom.

#### 2.2.1. Brain, Skin, Tumor and Glandular Phantoms

Skin, tumor, and average brain phantoms were prepared using the same ingredients (distilled water (DI), gelatine, sunflower oil, and dishwashing liquid) in different amounts. Furthermore, in the preparation of skin and tumor phantoms, the DI water was heated only to 65 °C, whereas in the preparation of the brain phantom, the water was heated to 85 °C.

**Figure 1:**
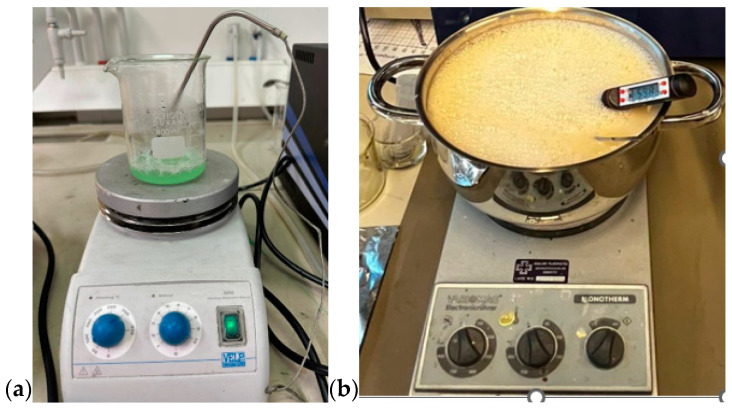
(**a**) Preparing tumor phantom on a beaker, (**b**) preparing brain phantom in a large kettle, with careful control of the temperature.

First, all the ingredients were measured separately using a high-precision scale. Gelatine was added to distilled water and the mixture was heated slowly till 65 °C (for the skin and tumor phantom) while stirring on a magnetic hot-plate stirrer. Sunflower oil was heated separately until 65 °C before it was added to the water-gelatine mixture, together with dishwashing liquid. In glandular phantom, sugar is used instead of oil and dishwashing liquid. The stirring continued for around five minutes. The mixture was then removed from the hot plate while being continuously stirred, and the heat was turned off, allowing the temperature to drop to about 50 °C. The mixture was poured on the mold for polymerization. The molds are described and illustrated in Section 2.3. For tumor phantoms and smaller skin-tissue phantoms, a beaker of size 0.5 L is sufficient (Figure 1a), but for realistic-sized brain phantoms, a large kettle is needed (Figure 1b). Stirring should be done gently to avoid the formation of excessive air bubbles, which affect dielectric properties. Air bubbles can also be removed from the phantom surface before solidification.

#### 2.2.2. Muscle and Intestinal Phantoms

The procedure for preparing the muscle phantom mixture is similar to that used to prepare the skin phantom, except that NaCl is added to the DI water before the gelatin is added. This muscle phantom recipe is commonly used for intestinal phantoms because the dielectric properties of muscle and intestines are similar. When a layered muscle phantom is prepared, the phantom mixture is poured onto a tray of size 40 cm × 40 cm for solidification. The tray is filled to a depth of 21 mm to obtain a muscle layer 20 mm in thickness, as in this study case, the phantom shrinks by approximately 4% during the solidification process. Intestinal phantoms can be prepared as layered phantoms or as realistic-shaped phantoms, which are described in Section 2.3.3.

#### 2.2.3. Fat Phantoms

There are several fat phantom recipes in the literature, but due to the high amount of sunflower oil used, the polymerized phantoms are fatty, wet, and break easily, making them difficult to use with 3D phantom models. These challenges with existing phantom recipes encouraged us to develop a more solid fat phantom using pure propylene glycol, which was presented for the first time in [51]. This paper briefly summarizes the procedure steps. Firstly, gelatine was added to distilled water on a hot-plate stirrer, and the mixture was heated slowly to 65 °C while stirring for 5 min. Propylene glycol was heated separately to around 50 °C and then added to the gelatin-DI water mixture, which was continuously stirred until the solution reached 65 °C. Then, xanthan gum was added to the solution and stirred in thoroughly. Finally, dishwashing liquid was added and mixed well into the solution. The mixture was poured into an appropriate mold and refrigerated for 24 h.

#### 2.2.4. Measurement of Dielectric Properties of Phantoms

**Figure 2:**
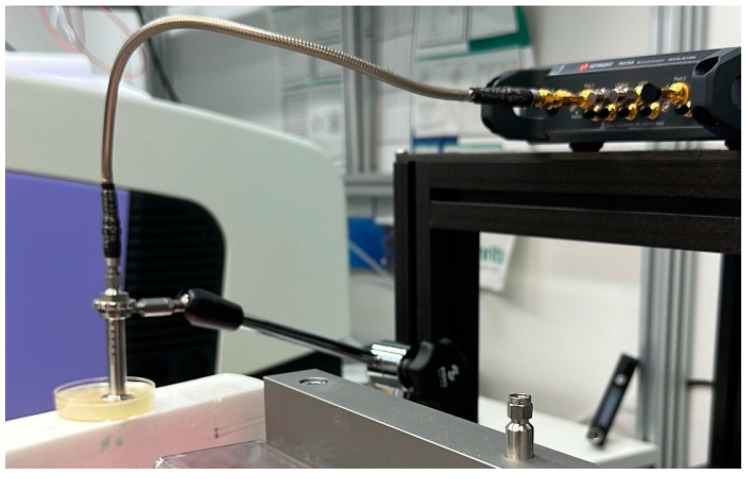
System used to measure the dielectric properties of phantoms with a VNA and a SPEAG probe.

The dielectric properties of the phantoms were measured using a Vector Networks Analyzer (VNA) 8720ES connected to SPEAG’s Dielectric Assessment Kit (DAK). The DAK 3.5 software converts the measured complex *S11* of the phantom sample into the complex permittivity and conductivity. The operational frequency range of this application is 200 MHz to 20 GHz, with a sweep of 117 points. The device was calibrated using the “Head” model from Speag’s calibration kit before each set of measurements to maintain accuracy. The dielectric properties of the sample were measured twice at three different locations, and the average value was calculated from these samples. The measurement system used for measuring the dielectric properties of the phantom sample is presented in Figure 2.

#### 2.2.5. Verification of Phantoms with EM-Simulations

In [51], we presented a novel idea for the verification of phantoms using tissue-layer models in electromagnetic simulations for fat phantoms. Firstly, the antenna reflection coefficient (*S11* parameter) was calculated for reference cases using actual dielectric values of biological tissue in tissue-layer models. Thereafter, these reference *S11* parameters were compared with *S11* parameters calculated by using dielectric values of the prepared phantom in the same tissue-layer models. This comparison aims to visualize the effect of small differences in the dielectric properties of the phantoms on the simulated antenna reflection coefficients. The comparative results provide deeper insights into antenna performance in realistic cases with phantom-based trials. This idea is further elaborated and extended in this paper to verify both the *S11* parameter and the channel transfer function (*S21*) parameters for all the presented phantom models.

**Figure 3:**
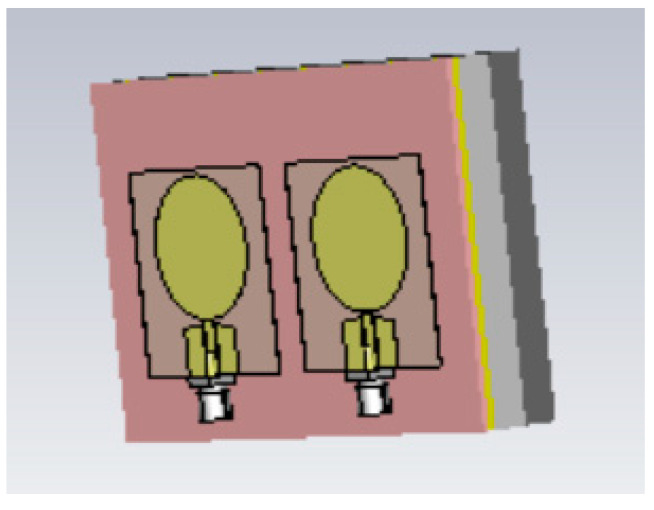
The tissue-layer simulation model.

The simulations were conducted using the electromagnetic simulation software Simulia Dassault CST Studio Suite 2023 [54]. Two different layer models were used in the simulations for different phantom verifications: Layer Model 1, which resembles the head model, to verify skin, fat, and brain phantoms; and Layer Model 2, which resembles the abdominal area, to verify muscle and intestinal phantoms. Layer Model 1 with on-body antennas is presented in Figure 3. The thicknesses of the tissue layers in both layer models are presented in Table 3.

For the simulations, human tissue values were automatically fetched from CST’s BioModel materials library. These values were used for the reference case simulations. Further, to test the developed phantom models, the loss tangent (tan⁡δ) values for the phantom cases were calculated from the measured conductivity values using the formula

$$\tan\delta =\frac{\sigma }{{\omega \varepsilon }_{0}\varepsilon_{r}}$$

where σ is the conductivity in S/m; ω=2πf with *f* the evaluated frequency in Hz; $\varepsilon_{0}=8.854$e^−12^ is the free space permittivity; and $\varepsilon_{r}$ is the real part of the complex permittivity value [55]. The obtained tan⁡δ values were inserted into CST, and simulations were carried out to validate the impact of the differences in dielectric properties on the *S11* and *S21* parameters. A close match between both the *S11* curve and the *S21* curve from the reference cases and the phantoms showcases that the developed phantoms have similar antenna reflection coefficients compared to real human tissue. This observation also reinforces the fact that developed phantoms have the same dielectric properties as actual biological tissues. The on-body antenna utilized in this work is an improved version of the flexible UWB antenna designed for wearable health-monitoring applications [56]. It is slightly larger than the antenna presented in [56], with a size of 40 mm × 40 mm × 0.125 mm, but it has better radiation characteristics in terms of gain towards the body. The implanted antenna is the same as that used in [57].

### 2.3. Phantom Molds for Realistic 3D Emulation Platforms

In this section, phantom molds, as well as procedures to develop phantoms with realistic shapes, are presented.

#### 2.3.1. Brain Mold

**Figure 4:**
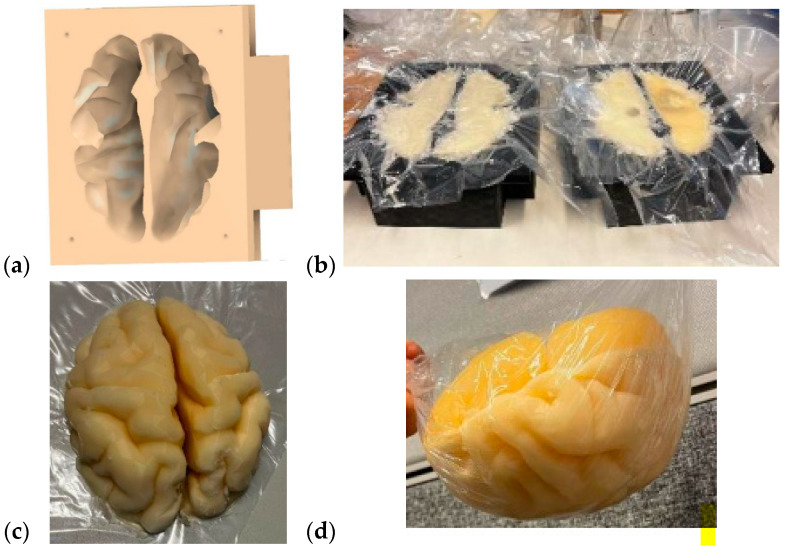
(**a**) A negative model of the brain phantom, (**b**) lower (left) and upper (right) molds of the brain after the phantom mixture was poured for solidification, (**c**) solidified upper part of the brain phantom, (**d**) whole brain phantom (upside down).

The brain mold was originally obtained from a printable 3D brain mold from [58]. The mold resembles an average brain in size, but it can be scaled for different sizes. Next, the negation of the 3D model was performed with the Fusion360 program to yield a 3D brain mold model, as illustrated in Figure 4a. The mold is divided into two pieces: the upper part and the lower part of the brain. Next, the 3D printing of the molds was carried out with a 3D printer, a process that takes approximately 24 h for an average-sized brain.

The brain-phantom mixture was poured into the molds, as shown in Figure 4b, with the lower part and upper parts of the brain phantom on the left and right, respectively. For brain-tumor-detection studies, the previously made, well-solidified tumor is set inside the brain-phantom mixture before solidification. However, the brain-phantom mixture should be cooled before the tumor phantoms are inserted to avoid melting the tumor. Approximately two days are needed for the brain phantoms to become fully solidified. Figure 4c presents the solidified upper part of the brain phantom, and Figure 4d is the full brain phantom (from an upside-down view). For brain-tumor-detection studies, it is essential to produce a reference brain phantom without tumors using an identical brain mold. The use of tumorous and reference brain phantoms that are identical in size and shape allows realistic and reliable investigations, e.g., of how tumors change signal propagation inside the brain tissue.

#### 2.3.2. Breast Phantoms for Breast-Tumor-Detection Studies

**Figure 5:**
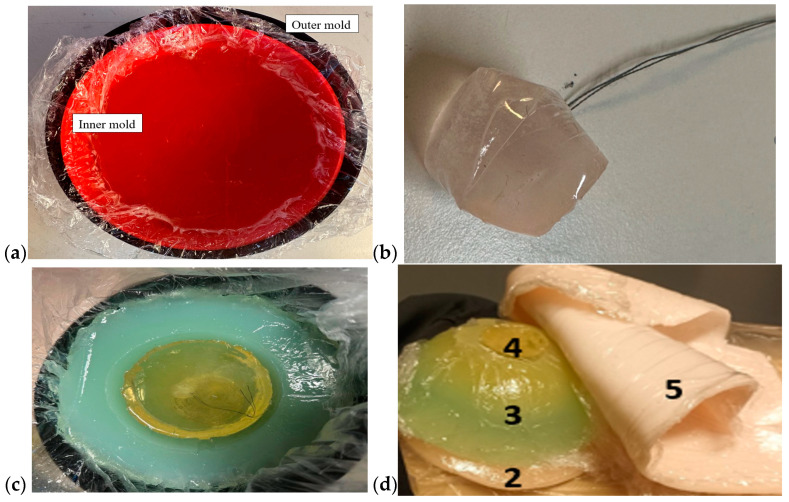
(**a**) Outer mold for fat tissue, inner mold for glandular tissue, (**b**) tumor phantom, (**c**) fat phantom with a tumorous glandular phantom inside, and (**d**) the realistic phantom emulation platform for breast-cancer-detection studies with a muscle phantom (2), fat phantom (3), glandular-tissue phantom (4), and skin phantom (5).

Breast phantoms were built separately from skin, fat, glandular tissue, and muscle tissues. In this case, the molds were simpler: the fat mold was a simple bowl with a diameter of 18 cm, and the glandular-tissue mold was a smaller bowl with a size depending on the targeted breast density, as shown in Figure 5a. The glandular-tissue mold was placed inside the fat-tissue mold before a liquid fat-tissue mixture was poured into the mold for solidification. The fat tissue was solidified in a form such that different glandular phantoms (reference and tumor tissues) could be easily interchanged.

Also, in this case, two glandular tissues were prepared: one with a tumor and another, identical in size, without the tumor, for the reference case. Tumors of different sizes were prepared with a thread attached, as shown in Figure 5b, to ease the addition of the tumor to the glandular tissue during the solidification process (Figure 5c). Additionally, skin and muscle-tissue phantoms were prepared for the final setup. For the skin phantom, a simple tray 40 cm × 40 cm in size, covered with thin plastic, was used as a mold. The muscle tissue was prepared on a smaller plane tray, 20 cm × 20 cm. Figure 5d presents the realistic phantom emulation platform used in breast-cancer-detection studies.

#### 2.3.3. Abdominal Molds

A realistic phantom setup for the abdominal area consists of skin, fat, muscle, visceral fat, and intestinal phantom layers. In this scenario, skin, outer fat, and muscle layers were modeled as pure layer models; hence, simple trays of varying sizes were used as molds. In this example scenario, the skin thickness was 1.5 mm and the muscle thickness was 15 mm. Additionally, liquid propylene glycol was used as a fat phantom, as proposed in [51]. The liquid propylene glycol was poured into a plastic bag, which was sealed with a heat sealer for the targeted size. With a liquid phantom, the fat-tissue thickness can be easily adjusted according to the study scenarios; in this example case, it was 20 mm.

**Figure 6:**
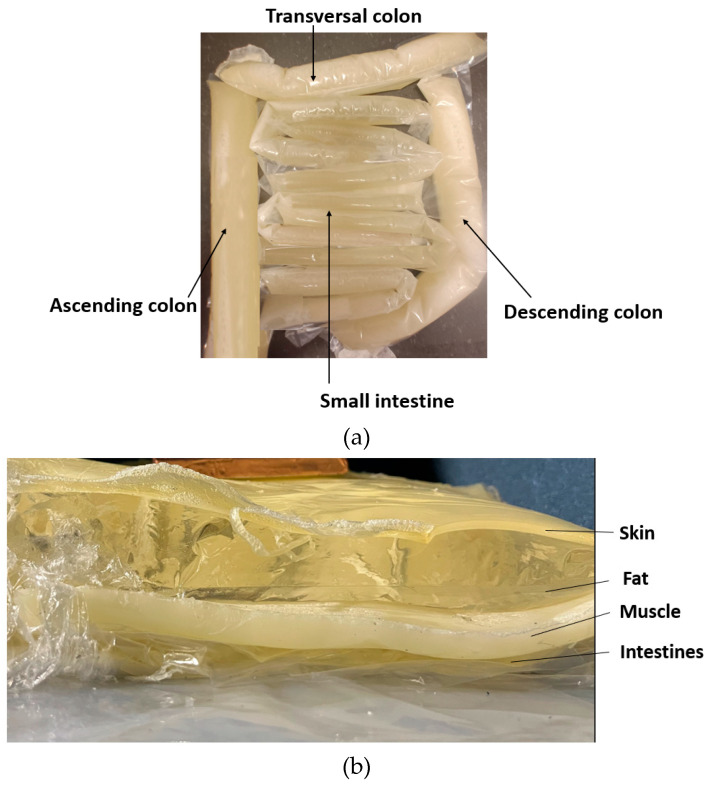
(**a**) small and large intestines, made in realistic sizes, (**b**) layered setup including skin, fat, muscle, and intestine layers.

As the shape of the intestinal area is complex and variable, the phantoms resembling small and large intestines with realistic shapes were developed by formulating molds from plastic using a heat sealer. The thickness of the small-intestine plastic mold was 2.5 cm, and the mold for the colon was 4 cm thick [59]. The plastic intestine molds were filled with a phantom mixture before solidification. The phantom mixture must be poured carefully to avoid the creation of excessive air bubbles. Typically, some bubbles appear, especially when the phantom mixture is poured into the small-intestine molds, which were thinner than the colon molds, but most of the air bubbles can be removed by carefully transferring the air bubbles to the upper part of the mold and carefully removing them with a spoon before sealing the mold. Additionally, the air bubbles can also be removed after sealing by carefully pressing the mold. Next, the molds were formed into the appropriate shape to resemble small and large intestine structures, as shown in Figure 6a, and were left for solidification until the following day.

The full phantom emulation platform is illustrated in Figure 6b. This kind of measurement setup is useful, e.g., in realistic radio-channel evaluations for capsule endoscopy because the measurement setup makes it possible to verify the results obtained in the simulations using anatomical voxel models [60]. Additionally, this kind of system is useful in general in studies relating to the detection of abnormalities in the intestinal region.

## 3. Results

The Results section is divided into three Subsections: Section 3.1 presents evaluations of the impact of the phantoms’ cooking temperatures on the dielectric properties of skin and fat phantoms; Section 3.2 presents the results of dielectric-property measurements for skin, muscle, glandular, brain, and fat tissues. The evolution of the recipes is shown by presenting the dielectric properties of the results of different recipe trials. Additionally, the longevity of each recipe is evaluated. Finally, the results of phantom verification from electromagnetic-layer-model simulations for different scenarios are presented in Section 3.3.

### 3.1. Effect of Cooking Temperature on the Dielectric Properties of Phantom Recipies

The particular heating temperature used for the preparation of a phantom is often debated within the research community. Therefore, in this section, different recipes are analyzed to study the effect of cooking temperature on the dielectric properties of the phantom. The muscle, skin, and fat phantom recipes were prepared with cooking temperatures of 65 °C, 75 °C, and 85 °C and then analyzed. It is to be noted that the original recipe for the skin phantom specifies cooking at 65 °C.

**Figure 7:**
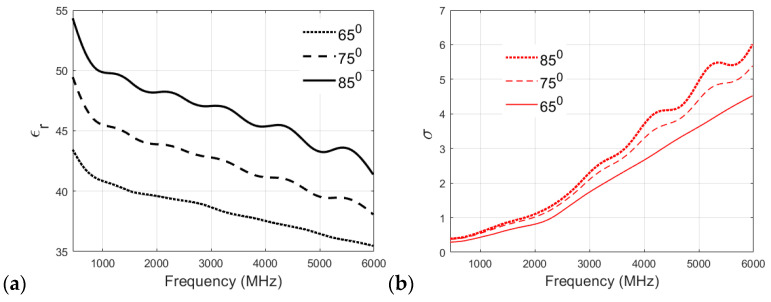
(**a**) Relative permittivity and (**b**) conductivity (S/m) v/s frequency (MHz) plots for skin phantom at 65 °C, 75 °C, and 85 °C.

Figure 7a,b showcases the effect of cooking temperature on the relative permittivity and conductivity of the skin phantom. The relative permittivity clearly increases with the cooking temperature, as can be visualized from Figure 7a, which plots the permittivity v/s frequency values for the three cooking temperatures for the skin phantom. Again, the original recipe, cooked at 65 °C, shows a smaller reduction in permittivity values with increasing frequency compared to 75 °C and 85 °C recipes, which proves the suitability of the proposed recipe for the skin phantom. It can be visualized from Figure 7b that the conductivity of the skin phantom increases with an increase in observation frequency for all three recipes at 65 °C, 75 °C, and 85 °C, but the phantom recipe cooked at 65 °C shows a smooth and almost linear transition in conductivity values, whereas some oscillations are observed in the conductivity values of the phantoms cooked at 75 °C and 85 °C.

**Figure 8:**
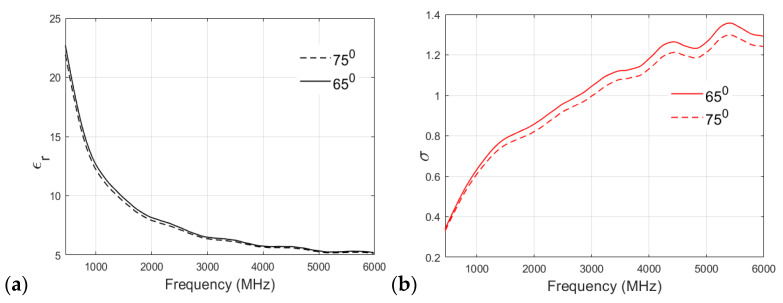
(**a**) Relative permittivity and (**b**) conductivity (S/m) v/s frequency (MHz) plot for fat phantom at 65 °C and 75 °C.

A similar analysis of the fat phantom cooked at 65 °C and 75 °C is shown in Figure 8a,b for relative permittivity and conductivity, respectively. As shown in Figure 8a, an interesting observation can be drawn from the permittivity v/s frequency plot: there is no significant effect of cooking temperature on the relative permittivity of the fat phantom, and both recipes show negligible differences in permittivity values at higher frequency bands. Figure 8b shows that both of the recipes have similar conductivity values at lower frequencies. For example, at 500 MHz, the conductivity value observed for the 65 °C sample was 0.3608 S/m, whereas that of the 75 °C sample was 0.3497 S/m. However, there is a clear distinction between conductivity values at the higher frequency band (>2 GHz). In the case of the fat phantom, a cooking temperature of 85 °C was not possible because the mixture became too grainy.

### 3.2. Evaluation of Dielectric Properties of Phantoms

The phantoms underwent development through various trial iterations, during which their dielectric properties, i.e., relative permittivity and conductivity—were measured at different frequencies. In this Section, evolution of skin and tumor phan-tom recipes is described. Furthermore, the longevity of different tissue phantoms was assessed by measuring their dielectric properties after specific time intervals: 5 h, 24 h, 1 week, and 10 days.

#### 3.2.1. Measurement Analysis and Summary for Skin and Tumor Phantoms

The skin and tumor phantoms were developed through a series of rigorous trials and fine-tuning of ingredient concentrations. Different recipe trials are presented in Table 4 and Table 5 for skin and tumor phantoms, respectively. For tumor phantoms, multiple experiments had to be conducted to obtain characteristics that correspond to actual tumor tissue.

The initial tumor phantom trial, labeled TS1, comprised distilled water, gelatine, sunflower oil, and dishwashing liquid. However, the relative permittivity values for TS1 fell significantly below those of real tumor tissue, highlighting the necessity for further adjustments. Moreover, the mixture exhibited an excessively oily and liquid consistency, rendering it unable to solidify properly. In the subsequent trial, TS2, efforts were made to address the issue by reducing the water content to encourage solidification. However, despite these adjustments, the mixture continued to exhibit excessive oiliness. Although TS2 demonstrated a decrease in relative permittivity compared to TS1, it failed to resolve the consistency problem. To further improve the mixture, trials ranging from TS3 to TS8 were conducted. These experiments involved decreasing the oil content slightly while also reducing the gelatine and increasing the water content in each trial. These modifications resulted in a gradual rise in relative permittivity values. Notably, TS7, which contained 20.3 mL of distilled water, closely mirrored the relative permittivity and conductivity values of actual tumor tissue. Furthermore, TS7 successfully achieved the desired solid consistency. Based on the results obtained from the tumor-phantom trials, the optimal composition was determined to be 20.3 mL of distilled water, 1.63 g of gelatine, 1.1 mL of sunflower oil, and 0.9 mL of dishwashing liquid. This specific combination closely mimicked the dielectric and physical properties of actual tumor tissue.

For the skin phantoms, several trials were conducted (as detailed in Table 3), and the optimal composition was found to be 10 mL of distilled water, 3.01 g of gelatin, 1.68 mL of sunflower oil, and 0.83 mL of dishwashing liquid. The prepared skin and tumor phantoms were then measured at different time intervals at 2.5 GHz and 6 GHz. Remarkably, they remained stable and reliable for up to one week, as indicated in Table 4. This durability allows for extended experimentation and analysis without significant alterations in their dielectric properties.

In summary, the developed skin and tumor phantom composition closely mimics the properties of the respective tissues, enabling precise and controlled experiments. A more detailed description of the phantom trials can be found in [52].

#### 3.2.2. Glandular Phantom Longevity

The glandular phantom is prepared with the recipe presented in Table 2. Longevity evaluations are carried out by measuring dielectric properties after 5 h, 24 h, 7 days, and 10 days after preparation. The evaluation results presented in Table 6 reveal that this phantom maintains its properties for up to 10 days, especially at 6 GHz and 8 GHz [52]. Instead at 2.5 GHz, relative permittivity changes slightly. Changes in conductivity are small within the evaluated frequency ranges.

#### 3.2.3. Muscle Longevity

Muscle phantom is prepared with the recipe presented in Table 2. The dielectric properties of the developed muscle phantom correspond closely to those of real muscle tissue, as shown in Table 7. Table 7 also presents the dielectric properties after 5 h, 24 h, 7 days, and 10 days after the development. Longevity evaluations show that muscle phantom remains usable for approximately 7 days. After 7 days, the relative permittivity starts to decrease as the water evaporates gradually from the phantom. At the same time, the conductivity increases [52].

#### 3.2.4. Fat Phantom

The fat phantom, its evolution over different trials, and its longevity were studied in detail in [51], and thus this paper only summarizes the values of the best recipe in Table 8. In [51], it was emphasized that the final trial (trial 14) had the most realistic dielectric properties, but the physical characteristics of the phantom were not sufficiently solid to be used in 3D molds.

### 3.3. Verification of the Final Phantom Recipes with EM Simulations

In this section, the phantom models proposed in this work are verified using the validation method presented in Section 2.2.5. Each phantom was first evaluated individually by changing the dielectric properties of individual tissues i.e., skin, brain, muscle, fat, and small intestine separately (Section 3.3.1, Section 3.3.2, Section 3.3.3, Section 3.3.4 and Section 3.3.5). The evaluations of the skin and brain phantoms were carried out with layer model 1 (LM1), which represents the head tissue, and an on-body antenna setup. The muscle, fat, and SI phantoms are evaluated with layer model 2 (LM2), which resembles abdominal tissues, and an on-body implant antenna setup. In each case, the results are compared with the reference values generated using actual values from biological samples. Finally, the whole LM2 phantom setup was evaluated by changing the dielectric properties of each tissue layer to those obtained with the developed phantoms and comparing the results using reference LM2 (Section 3.3.1, Section 3.3.2, Section 3.3.3, Section 3.3.4 and Section 3.3.5).

#### 3.3.1. Verification of the Skin Phantom

**Figure 9:**
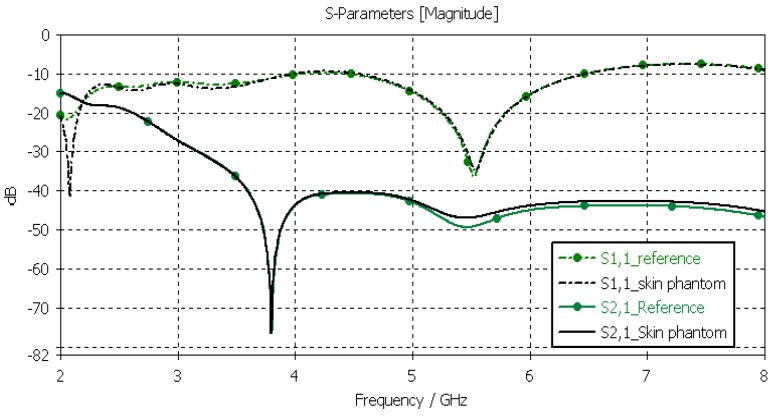
Skin-phantom verification with *S11* and *S21* simulation using layer model 1. In the phantom case, the dielectric properties of the tissue layers are the same as those presented in Table 2 for the skin phantom. In the reference case, the dielectric properties are the same as those of average human tissue [53].

The skin phantoms were evaluated with Layer Model 1 with two flexible on-body antennas. In this case, only the dielectric properties of the skin were changed, while the dielectric properties of the other tissues were kept to the reference values retrieved from [53]. The values of *S11* and *S21* for the reference case and skin phantom case are shown in Figure 9. As can be seen, the difference between the skin phantom and reference cases is negligible within most of the simulated frequency band; the maximum differences can be found at 5.5 GHz: 1.4 dB for *S11* and 2 dB for *S21* results.

#### 3.3.2. Verification of the Brain Phantom

**Figure 10:**
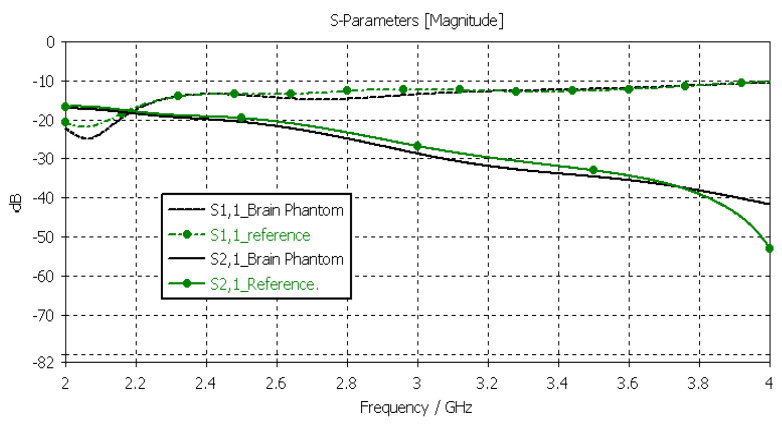
Verification of the brain phantom with *S11* and *S21* simulations using layer model 1. In the phantom case, the dielectric properties of the tissue layers are the same as those presented in Table 2 for the brain phantom. In the reference case, the dielectric properties are the same as those of average human tissue [53].

Next, the brain phantom was evaluated using Layer Model 1 and flexible antennas. In this case, only the dielectric properties of the brain were changed, and the dielectric properties of the other tissues are kept to the reference values retrieved from [53]. The results are presented in Figure 10. In the case of brain phantoms, the differences in *S11* results are somewhat similar to those found for the skin phantoms: the maximum difference was 2 dB, and it occurred at 2.1 GHz. Instead, there are clearer differences in the *S21* results, especially at 2.8–3.6 GHz and at 4 GHz. The maximum differences in these ranges are 2 dB and 10 dB. However, *S21* analysis for frequency ranges above 4 GHz is seldom used due to high propagation loss, and thus, this phantom recipe can be considered suitable for different applications.

#### 3.3.3. Verification of Muscle and Intestinal Phantoms

**Figure 11:**
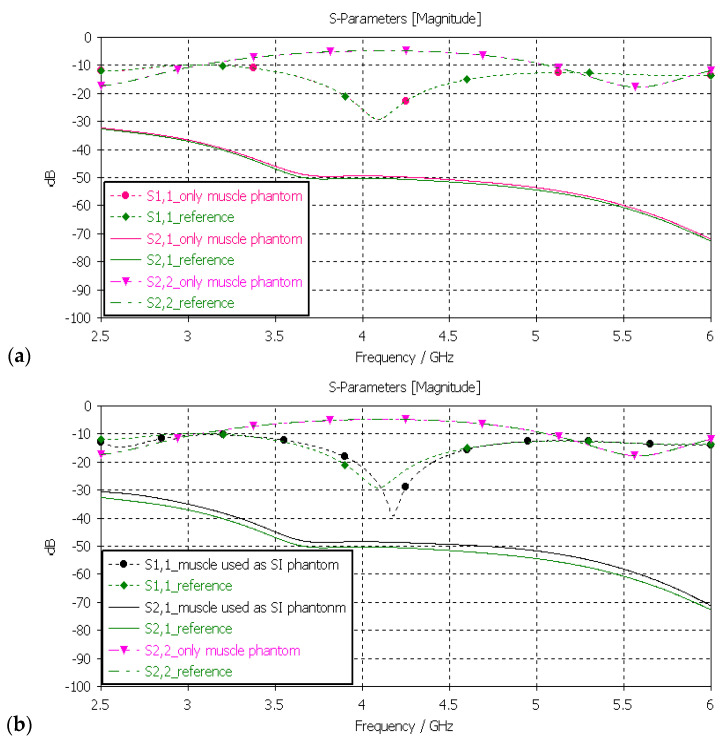
(**a**) Verification of muscle and (**b**) intestinal phantoms with *S11* and *S21* simulations using layer model 2. In the phantom case, the dielectric properties of the tissue layers were the same as those presented in Table 2 for the muscle phantom. In the reference case, the dielectric properties were the same as those of average human tissue [53] for (**a**) muscle and (**b**) intestinal tissues.

Next, the muscle phantom was verified with layer model 2, which resembles abdominal tissue layers. In this case, the dielectric properties of the developed muscle phantom were used both in the muscle and intestinal layers, as is common in the literature [5]. The channel was evaluated between the implant and on-body flexible antennas. The evaluations were carried out separately only for the muscle layer and the small-intestine layer, as presented in Figure 11a,b, respectively.

In evaluation of the muscle layer shown in Figure 11a, changes in the *S11*, S22, and *S21* results between the reference and phantom cases were negligible. The maximum difference, only 1.5 dB, was observed in *S21* results at 5–6 GHz. Besides the non-significance of the difference, commonly considered frequency ranges for implant (ingestible) communications and sensing are at ISM band 2.5 GHz, the first part of the UWB band at 3.1–4.1 GHz, and 3.75–4.25 GHz [59,60,61]. In evaluations of the SI layer, presented in Figure 11b, the differences between the reference and phantom cases were small except in *S11* (implant antenna reflection coefficient) at around 4.2 GHz, were the difference was as large as 13 dB. This difference was due to the fact that the small-intestine and muscle layers had small differences in their dielectric properties, as shown in Table 2. Nevertheless, the differences in *S21* results were relatively small, reaching a maximum of 2 dB at 5 GHz.

#### 3.3.4. Verification of the Fat Phantom

**Figure 12:**
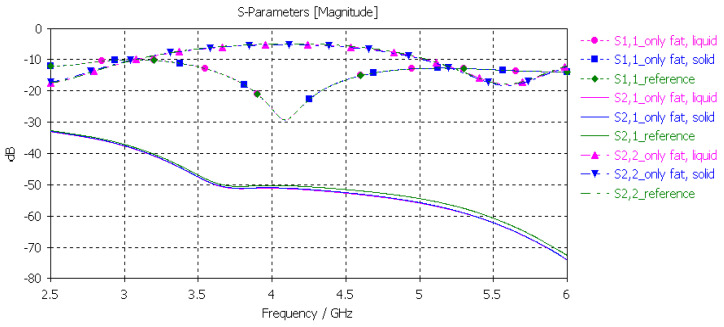
Verification of solid and liquid fat phantoms with *S11* and *S21* simulations using layer model 2. In the phantom case, the dielectric properties of the tissue layers were the same as those obtained in phantom measurements, as presented in Table 2. In the reference case, the dielectric properties were same as those of average human tissue [53].

Finally, the fat phantom was evaluated with layer model 2, a flexible on-body antenna, and the capsule antenna, using the dielectric properties of both liquid and solid fat phantoms. The results, shown in Figure 12, show that the differences between the liquid and solid phantoms were minor compared to the reference case: the maximum difference was 2 dB at 5.5 GHz.

#### 3.3.5. Verification of the Full Abdominal Phantom Layer Model

**Figure 13:**
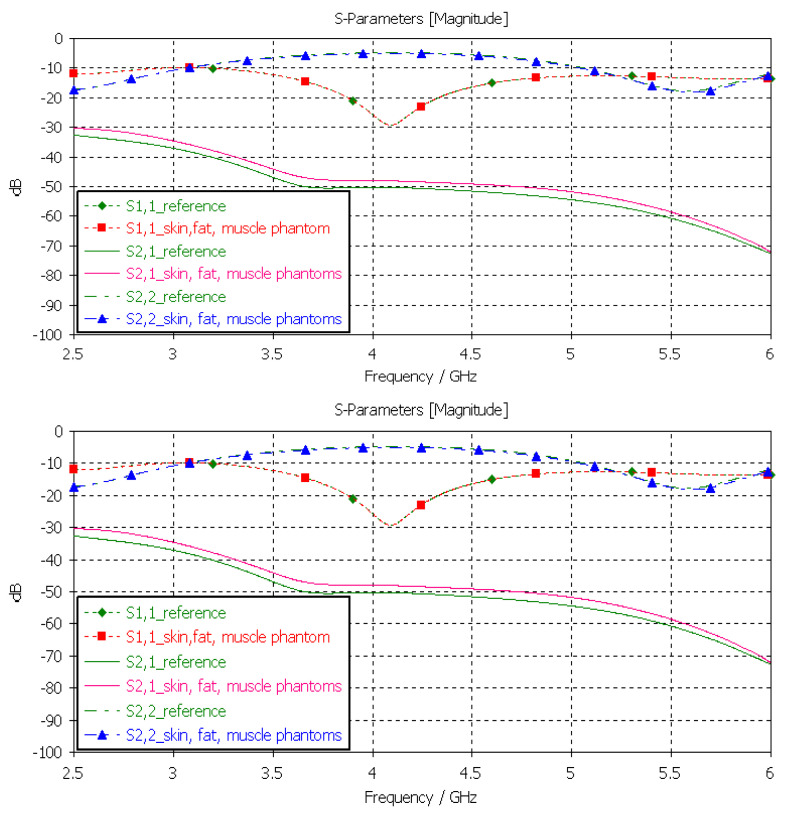
Verification of solid and liquid fat phantoms with *S11* and *S21* simulations using layer model 2. In the phantom case, the dielectric properties of the tissue layers were the same as those obtained in phantom measurements presented in Table 2. In the reference case, the dielectric properties are the same as those of average human tissue [53].

Finally, the full abdominal layer model was evaluated using skin, fat and muscle phantoms. Muscle phantoms were also used in the small-intestine layer. The results are presented in Figure 12. As can be seen, the results are similar to those presented with SI evaluations in Figure 13, with only minor changes in *S21* results. To obtain even better accuracy, the muscle phantom could be further developed to resemble the small intestine by increasing the amount of water and salt slightly to increase permittivity and conductivity, respectively.

The differences between the *S11*, S22, and *S21* parameters obtained with the simulations conducted using the dielectric properties of the phantoms and reference cases for each tissue at frequencies of 2.5 and 6 GHz are shown in Table 2. Additionally, the maximum difference at the corresponding frequency is included. As can be seen, although there are differences between the dielectric properties of the developed phantoms and those of real human tissues, the differences in terms of their use in practical scenarios are not highly significant. Therefore, it can be concluded that the presented phantoms are valid both for on-body and in-body sensing/communications applications.

## 4. Discussion

The results obtained from the tissue phantom verifications show that although there might be some differences in the measured dielectric properties of the developed phantoms with respect to those of real human tissues, the differences in *S11* and *S21* simulation results are not highly significant. Especially in the frequency ranges targeted for medical applications, such as the 2.5 GHz ISM band and the UWB band, the differences are minor. Therefore, it can be concluded that the presented phantoms are valid both for on-body and in-body sensing/communications applications.

The evaluations of the impact of the phantom cooking temperature validate the importance of carefulness with temperature during phantom cooking. Depending on the phantom, the dielectric properties may change significantly if the temperature increases excessively. With the skin phantom, the increase was a maximum of 13 units in relative permittivity and 1.5 S/m in conductivity. The changes in relative permittivity, while larger, were somewhat similar across the whole measured bandwidth. By contrast, with conductivity, the changes were smaller at lower frequencies than at higher frequencies. This result is new, interesting, and useful for phantom development. Besides emphasizing the need for carefulness in phantom preparation, this result also enables the use of same recipe for different tissue phantoms if different cooking temperatures are used. Interestingly, with the fat phantom, the changes due to cooking temperature are clearly minor. As a future work, the authors will study the impact of temperature on other phantom recipes as well.

In general, the results presented in this paper provide several new insights for use in the microwave-sensing field. This paper presents new phantom recipes and new innovative approaches to the preparation of realistic 3D phantoms for the validation of several emerging medical monitoring applications. In future work, we will present comprehensive evaluations of several monitoring applications using the proposed realistic platforms. New phantom recipes and new innovative approaches to preparing realistic 3D phantoms facilitate the introduction and validation of several novel medical-monitoring applications utilizing microwave techniques. These novel 3D phantoms can be considered part of the development of digital twins for healthcare applications.

## Figures and Tables

**Table 1:** Relative permittivity values at 2 GHz, 4 GHz, 6 GHz, and 8 GHz [53].

Tissue	Frequency			
	2 GHz	4 GHz	6 GHz	8 GHz
Brain (gray matter)	49.7	46.6	43.7	40.9
Brain (white matter)	36.7	34.5	32.4	30.4
Brain tumor	59.0	55.7	52.2	48.6
Fat	5.33	5.12	4.84	4.46
Glandular tissue	58.1	54.9	51.7	48.4
Breast tumor	63.0	59.1	56.6	55.4
Skin	38.6	36.6	34.9	33.2
Muscle	53.3	50.8	48.2	45.5
Large intestine	54.7	51.3	48.1	45.0
Large intestine lumen	53.3	50.8	48.2	45.5
Small intestine	55.4	51.6	48.3	45.1
Small intestine lumen	53.3	50.8	48.2	45.5

**Table 2:** Ingredients for different human-tissue phantom recipes.

Phantom Type	Concentration of Ingredients							
	DI Water (mL)	Gelatine (g)	Sunflower Oil (mL)	DW Liquid ^1^ (mL)	Xanthan Gum (g)	PG ^2^ (mL)	Sugar (g)	NaCl (g)
Skin	10	3.01	1.68	0.83	-	-		
Tumor	20.3	1.63	1.1	0.9	-			
Brain	9	1.5	1.1	0.5	-			
Glandular tissue	25.2	5.05	-	-	-	-	0.525	-
Muscle/Intestine	20	6.02	3.36	1.67	1.67	-	-	-
Fat	3	2	-	0.5	1	50	-	-

^1^ DW liquid: Dish-washing liquid. ^2^ PG: Propylene glycol.

**Table 3:** Thicknesses of tissue layers in Layer Model 1 and Layer Model 2.

	Skin (mm)	Fat (mm)	Skull Bone (mm)	Brain (mm)	Muscle (mm)	Small Intestine (mm)
Layer model 1 (head)	1.2	1.2	7.5	7.5	-	-
Layer model 2 (abdomen)	2.2	10	-	-	8	20

**Table 4:** Different tumor and skin phantom mixtures used in trials, with their recipes [52].

Phantom Type	Sample Trial	Concentration of Ingredients			
		Water (mL)	Gelatin (g)	Oil (mL)	Dishwasher (mL)
Tumor	TS1	22	1.7	4.25	0.95
	TS2	8	1.7	4.25	0.95
	TS3	12.3	1.63	1.1	0.9
	TS4	14.3	1.63	1.1	0.9
	TS5	16.3	1.63	1.1	0.9
	TS6	18.3	1.63	1.1	0.9
	TS7	20.3	1.63	1.1	0.9
	TS8	22.3	1.63	1.1	0.9
Skin	SS1	6	3.01	1.68	0.83
	SS2	8	3.01	1.68	0.83
	SS3	10	3.01	1.68	0.83

**Table 5:** Different tumor and skin phantom mixtures used in trials and their dielectric properties after 5 h, 24 h, 1 week, and 10 days.

Phantom Type	Sample Trial	Relative Permittivity/Conductivity(S/m)							
		After 5 h		After 24 h		After 7 days		After 10 days	
		2.5 GHz	6 GHz	2.5 GHz	6 GHz	2.5 GHz	6 GHz	2.5 GHz	6 GHz
Tumor	TS1	43.2/1.38	38.6/4.97	41.06/1.08	36.61/4.26	40.8/1.36	36.96/4.99	38.06/1.56	33.3/4.32
	TS2	28.4/2.87	24.6/3.16	28.7/2.81	24.6/3.63	29.3/2.145	24.31/3.62	25.3/1.98	20.6/3.13
	TS3	31.7/1.23	29.2/3.9	31.2/1.12	29.7/4.11	30.9/1.3	28.2/4.16	21.2/1.31	18.5/4.8
	TS4	38.7/1.03	35.12/4.23	38.92/1.04	34.27/4.09	38.7/1.122	33.21/4.03	37.92/1.02	26.25/3.98
	TS5	42.27/1.24	37.51/4.52	43.12/1.32	39.21/5.0	42.27/1.43	38.13/5.1	39.18/1.13	31.42/4.82
	TS6	49/1.45	45.2/5.53	50.9/1.46	45.27/5.6	50.34/1.54	43.52/5.34	40.4/1.32	39.7/4.99
	TS7	62.8/1.68	59.0/6.32	62.9/1.69	57.2/6.52	61.01/1.48	56.47/6.13	57.3/1.21	48.6/5.82
	TS8	70.5/1.75	67.3/6.84	69.0/1.75	63.1/7.16	69.51/1.48	61.26/6.951	61.1/1.83	56.4/6.27
Skin	SS1	30.07/0.93	26.37/3.2	35.07/0.08	27.14/2.15	29.86/1.655	25.12/3.478	20.26/0.54	17.25/2.12
	SS2	35.1/1.34	31.7/3.36	38.9/1.07	32.8/3.12	41.39/1.72	31.96/5.38	23.5/1.82	18.7/2.28
	SS3	40.3/1.48	36.9/4.78	38.2/1.96	34.1/3.76	41.22/1.54	34.11/5.51	30.1/0.93	26.37/3.2

**Table 6:** Glandular phantom recipe and its dielectric properties after 5 h, 24 h, 7 days, and 10 days.

	Time	Relative Permittivity/Conductivity (S/m)			
Frequency (GHz)		5 h	24 h	7 days	10 days
2.5		62.03/2.03	61.82/2.15	62.45/2.15	57.1/2.0
6		50.95/8.23	50.78/8.03	51.44/8.36	50.7/8.34
8		44.75/12.8	45.39/11.58	48.11/12.25	44.99/12.96

**Table 7:** Muscle phantom’s dielectric properties after 5 h, 24 h, 7 days, and 10 days [52].

	Time	Relative Permittivity/Conductivity (S/m)			
Frequency (GHz)		5 h	24 h	7 days	10 days
2.5		54.98/1.75	55.1/1.8	54.79/1.91	51.84/3.67
6		48.9/5.63	48.59/5.399	47.99/5.12	45.1/7.63
8		45.7/8.2	45.34/8.6	45.1/8.59	43.3/10.2

**Table 8:** Dielectric properties (permittivity/conductivity (S/m)) of the fat phantom [51] at different frequencies.

	2 GHz	6 GHz	8 GHz
Fat	6.4/0.75	5.0/0.953	4.76/1.02

## Data Availability

All the data is shared in this paper.
